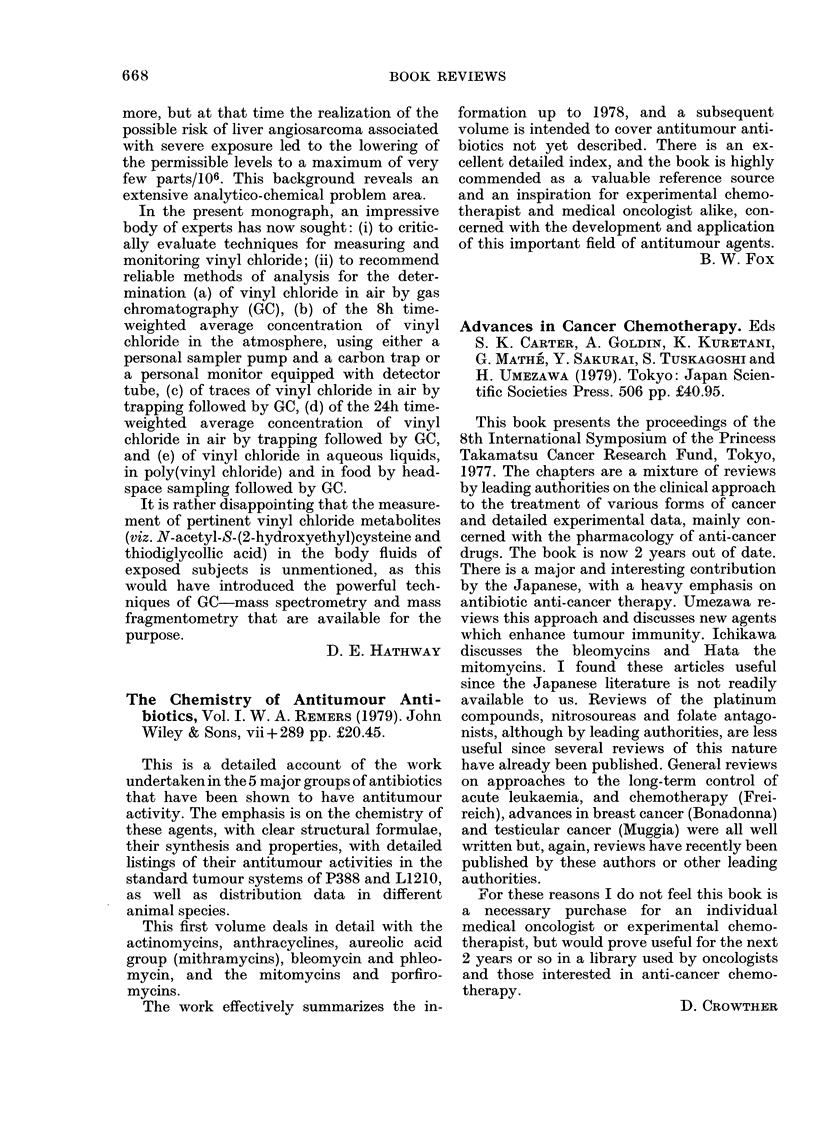# Advances in Cancer Chemotherapy

**Published:** 1979-10

**Authors:** D. Crowther


**Advances in Cancer Chemotherapy.** Eds S. K. Carter, A. Goldin, K. Kuretani, G. Mathé, Y. Sakurai, S. Tuskagoshi and H. Umezawa (1979). Tokyo: Japan Scientific Societies Press. 506 pp. £40.95.

This book presents the proceedings of the 8th International Symposium of the Princess Takamatsu Cancer Research Fund, Tokyo, 1977. The chapters are a mixture of reviews by leading authorities on the clinical approach to the treatment of various forms of cancer and detailed experimental data, mainly concerned with the pharmacology of anti-cancer drugs. The book is now 2 years out of date. There is a major and interesting contribution by the Japanese, with a heavy emphasis on antibiotic anti-cancer therapy. Umezawa reviews this approach and discusses new agents which enhance tumour immunity. Ichikawa discusses the bleomycins and Hata the mitomycins. I found these articles useful since the Japanese literature is not readily available to us. Reviews of the platinum compounds, nitrosoureas and folate antagonists, although by leading authorities, are less useful since several reviews of this nature have already been published. General reviews on approaches to the long-term control of acute leukaemia, and chemotherapy (Freireich), advances in breast cancer (Bonadonna) and testicular cancer (Muggia) were all well written but, again, reviews have recently been published by these authors or other leading authorities.

For these reasons I do not feel this book is a necessary purchase for an individual medical oncologist or experimental chemotherapist, but would prove useful for the next 2 years or so in a library used by oncologists and those interested in anti-cancer chemotherapy.

D. Crowther